# The family Plectopylidae (Gastropoda, Pulmonata) in Laos with the description of two new genera and a new species

**DOI:** 10.3897/zookeys.592.8118

**Published:** 2016-05-25

**Authors:** Barna Páll-Gergely, Igor V. Muratov, Takahiro Asami

**Affiliations:** 1 Department of Biology, Shinshu University, Matsumoto 390-8621, Japan; 2 KwaZulu-Natal Museum, P. Bag 9070, Pietermaritzburg 3200, South Africa; 3 School of Life Sciences, University of KwaZulu-Natal, Scottsville, 3206, South Africa

**Keywords:** Taxonomy, anatomy, radula, protoconch, new genus, new species

## Abstract

Previously only a single plectopylid species, *Helix laomontana* L. Pfeiffer, 1862 was reported from Laos. Here we erect *Naggsia* Páll-Gergely & Muratov, **gen. n.** for *Helix laomontana* based on the description of its reproductive anatomy and radula. Another species, *Hunyadiscus saurini* Páll-Gergely, **gen. & sp. n.** is described from Northern Laos based on conchological data. Helix (Plectopylis) andersoni Blanford, 1869, which is known from the Burmese-Chinese border region, is also classified within *Hunyadiscus* Páll-Gergely, **gen. n.** A third species, Gudeodiscus (Gudeodiscus) messageri raheemi Páll-Gergely & Hunyadi, 2015 is reported from Laos for the first time. The new localities represent the westernmost sites of the genus *Gudeodiscus*. The reproductive anatomy of the latter species is described.

## Introduction

Land snails of the family Plectopylidae are widely distributed in East Asia, from Nepal to southern Japan (Gude 1899b, Páll-Gergely and Hunyadi 2013, Páll-Gergely et al. 2015b). The taxonomic system of the Plectopylidae was established by Gude (1899a), who recognized seven sections (*Endothyra* Gude, 1899a = *Endothyrella* Zilch, 1960, *Chersaecia* Gude, 1899a, *Endoplon* Gude, 1899a, *Plectopylis* Benson, 1860, *Sinicola* Gude, 1899a, *Enteroplax* Gude, 1899a and *Sykesia* Gude, 1897b). Two of these (*Enteroplax* and *Sykesia* = *Ruthvenia* Gude, 1911) are now classified in different families (Páll-Gergely and Hunyadi 2013). Recent investigation resulted in the redefinition of Gude’s (1899a) genera as well as the description of three new genera (*Gudeodiscus* Páll-Gergely, 2013, *Halongella* Páll-Gergely, 2015 and *Sicradiscus* Páll-Gergely, 2013). With these, plectopylids are now classified in eight genera (Páll-Gergely and Hunyadi 2013, Páll-Gergely et al. 2015a, 2015b).

The single species reported from Laos to date was originally described as *Helix laomontana* L. Pfeiffer, 1862 (type locality: “Lao Mountains, Camboja”) and was classified in *Chersaecia* by Gude (1899a). Revision of *Endothyrella* showed that *Chersaecia* had been treated as a catchall category and that it contained species with very diverse conchological characters (Páll-Gergely et al. 2015b). We transferred five species from *Chersaecia* to *Endothyrella*, mainly based on the ribbed protoconch and the absence of an apertural fold. In *Chersaecia* we retained only species that were similar to the type species, *Plectopylis leiophis* Benson, 1860, in terms of the presence of an apertural fold and the finely granulated (not ribbed) embryonic whorls. *Helix laomontana*, has also been excluded from *Chersaecia*, but could not be included into any other existing genus. Live collected specimens allowed the clarification of the taxonomic position of *Helix laomontana*. Another species, namely Helix (Plectopylis) andersoni W. Blanford, 1869 was also excluded from *Chersaecia* (see Páll-Gergely et al. 2015b). Although no ethanol-preserved specimens were available for anatomical study, its shell characters provide sufficient information to clarify its systematic status. Additionally, we report two other species from Laos, one being new to science.

## Material and methods

Shell whorls were counted according to Kerney and Cameron (1979: 13) (precision 0.25).

For the nomenclature of lamellae (vertical parietal folds) and plicae (horizontal parietal folds and palatal folds) see Páll-Gergely et al. 2015b. Whenever possible, the internal lamellae and plicae were exposed by removing the shell wall at the appropriate part of the shells (inner view). If damaging the shells was not an option (too few shells available), the plicae were figured on the basis of their visibility through the shell wall (outer view). “Anterior” refers to the part or side of the armature closer to the aperture, “posterior” refers to the other side of the armature.

Ethanol-preserved specimens were dissected under a Leica stereomicroscope, equipped with a photographic camera. In the descriptions of the reproductive system, we used the terms “proximal” and “distal” in relation to the apical portion of the reproductive tract *i.e.* the ovotestis.

Individual buccal masses were removed and soaked in 2 M KOH solution for 5 hours before extracting the radula, which was later preserved in 70% ethanol. Radulae and shells were directly observed without coating under a low vacuum SEM (Miniscope TM-1000, Hitachi High-Technologies, Tokyo).

### Abbreviations

HNHM
Hungarian Natural History Museum (Budapest, Hungary) 


MNHN
Muséum National d’Histoire Naturelle (Paris, France) 


NHM
Natural History Museum (London, UK) 


NHMUK
 when citing registered specimens from the Natural History Museum (London, UK) 


NHMW
 Naturhistorisches Museum (Vienna, Austria) 


OK
 Collection Kenji Ohara, Nishinomiya Shell Museum (Nishinomiya, Japan) 


PGB
 Collection Barna Páll-Gergely (Mosonmagyaróvár, Hungary) 


RBINS
Royal Belgian Institute of Natural Sciences (Brussels, Belgium) 


RE
 Collection Reischütz (Horn, Austria) 


SMF
Senckenberg Forschungsinstitut und Naturmuseum (Frankfurt am Main, Germany) 


WM
 Collection Wim J. M. Maassen (Echt, The Netherlands) 


ZMH
Zoological Museum, Hamburg (Germany) 


## Taxonomic descriptions

### Family Plectopylidae Möllendorff, 1898

#### Gudeodiscus

Taxon classificationAnimaliaPulmonataPlectopylidae

Páll-Gergely, 2013

Gudeodiscus 2013 Gudeodiscus Páll-Gergely, In: Páll-Gergely and Hunyadi, Archiv für Molluskenkunde 142(1): 4, 8.

##### Type species.

*Plectopylis phlyaria* Mabille, 1887, by original designation.

#### Subgenus Gudeodiscus

Taxon classificationAnimaliaPulmonataPlectopylidae

Gudeodiscus 2015 Gudeodiscus (Gudeodiscus), — Páll-Gergely et al., ZooKeys 473: 13.

#### Gudeodiscus (Gudeodiscus) messageri raheemi

Taxon classificationAnimaliaPulmonataPlectopylidae

Páll-Gergely & Hunyadi, 2015

**Figure 1.**
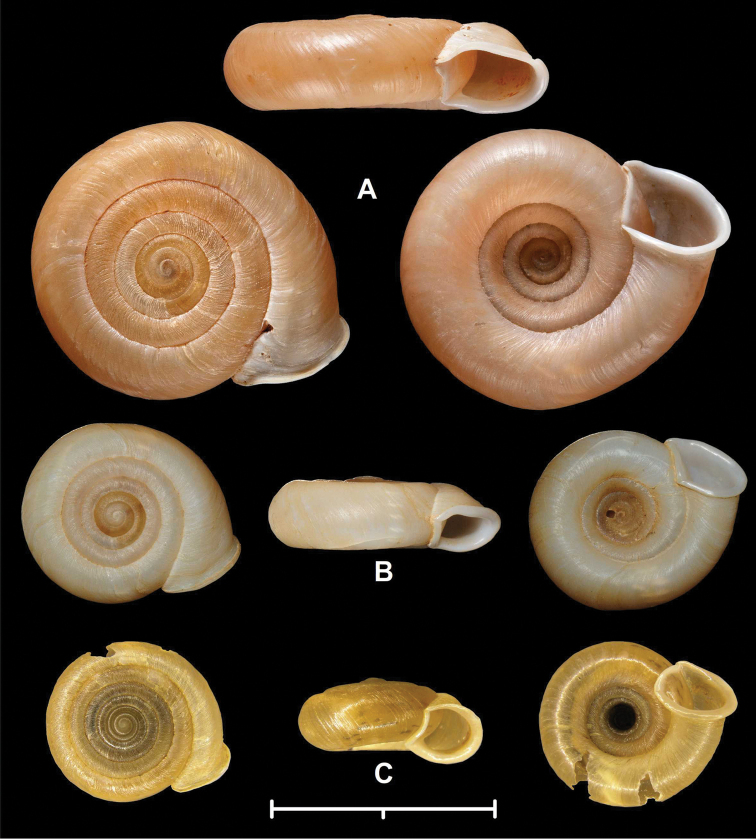
Shells of Plectopylidae species. **A** *Naggsia laomontana* (L. Pfeiffer, 1862) (syntype, NHMUK 20130004) **B** *Naggsia laomontana* (Laos, Luang Prabang Province, Ban Pak Ou, Nam Wu (opposite side of Ban Pak Ou), 364 m 20°03.48276'N, 102°12.79912'E, leg. Ohara, K. 13.10.2006., coll. PGB) **C** Gudeodiscus (Gudeodiscus) messageri raheemi Páll-Gergely & Hunyadi, 2015, locality code: 11L06. Photos: T. Deli (**B**), B. Páll-Gergely (**C**) and H. Taylor (**A**). Scale represents 20 mm.

**Figure 2.**
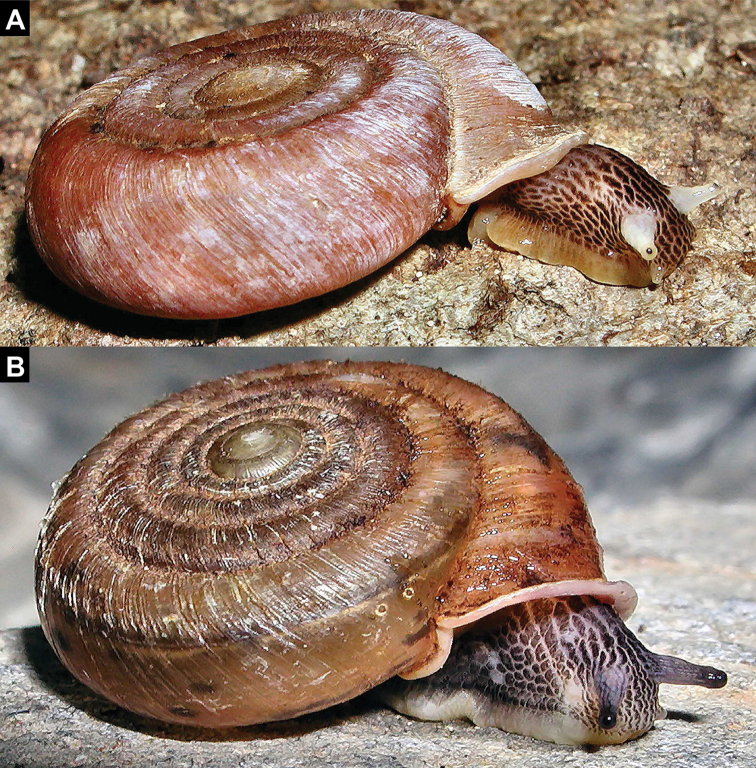
Living animals of Laotian Plectopylidae (anatomically examined specimens). A: *Naggsia laomontana* (L. Pfeiffer, 1862) **B** Gudeodiscus (Gudeodiscus) messageri raheemi Páll-Gergely & Hunyadi, 2015. Photos: Igor Muratov.

Figures 1C , 2B , 5G–H , 6 , 9D–G

Gudeodiscus (Gudeodiscus) messageri raheemi 2015a Gudeodiscus (Gudeodiscus) messageri raheemi, — Páll-Gergely et al., ZooKeys 473: 38–40, Figures 5D, 5E, 10A, 12R–V, 20, 28E, 29F–G, 31B, 35D–F.

##### Material examined.

**11L06** Laos, Luang Prabang Province, ca. 18 km SE of Muang Xiang Ngeun, on the left side of Nam Khan, limestone, black soil in limestone pockets, clay, under rocks and logs in old forest, 455 m a.s.l., 19°40.931'N, 102°19.743'E, leg. A. Abdou & I.V. Muratov, 30.10.2006, MNHN 2012-27054/38 shells (some of them are broken/juvenile) + anatomically examined specimen (Figs 1C, 2B, 5G–H, 6, 9D–G); **12L06** Laos, Luang Prabang Province, ca. 17 km SE of Muang Xiang Ngeun, on the left side of Nam Khan, limestone, black soil in limestone pockets, clay, under rocks and logs in old forest, 385 m a.s.l., 19°41.201'N, 102°19.197'E, leg. A. Abdou & I.V. Muratov, 30.10.2006, MNHN 2012-27055/21 shells (some of them are broken/juvenile); **34L06** Laos, Luang Prabang Province, ca. 6 km N of Phou Khoun, limestone, clay, under rocks in dry secondary forest under and above cliff, 1244 m a.s.l., 19°29.653'N, 102°24.470'E, leg. A. Abdou & I.V. Muratov, 16.11.2006, MNHN 2012-27056/11 shells (some of them are broken/juvenile); Laos, Vientiane Province, Tam Chang, Vang Vieng, leg. Pongrat Dumrongrojwattana, 11.06.2009., PGB/1, WM/1.

##### Reproductive anatomy.

A single living specimen collected in Laos, was killed by drowning and was stored in 70 % ethanol. A part of the body was extracted from the shell and was examined anatomically. The inner parts of the genitalia, such as the gametolytic sac, the diverticulum and the spermoviduct could not be extracted.

The reproductive anatomy of *Gudeodiscus messageri raheemi* was figured in the original description (Páll-Gergely et al. 2015a). The two anatomically examined Vietnamese specimens differed from each other mainly in the length of the penial caecum. The Laotian specimen possesses a short caecum, similar to one of the Vietnamese specimens. The length of the caecum probably has minor taxonomic value because it varies considerably within species (see also *Gudeodiscus phlyarius* in Páll-Gergely et al. 2015a). The inner wall of the epiphallus of the Vietnamese specimens had three, rather low, longitudinal folds. In contrast, the Laotian specimen had a single thickened fold internally.

##### Remarks.

Gudeodiscus (Gudeodiscus) messageri raheemi was described from Vietnam, where it inhabits the provinces Ninh Bình, Thanh Hóa, Sơn La, Hòa Bình and Nghệ An. The Laotian specimens agreed in shell morphology with the Vietnamese specimens. The new Laotian localities of *Gudeodiscus messageri raheemi* represent the westernmost record of the genus (Figure 14).

##### Ecology.

This taxon inhabits primary or old secondary broad-leaved forests, in the humid microenvironments under leaves, logs, limestone rocks and in black soil accumulated inside limestone pockets. The live collected specimen co-occured with *Garnieria mouhoti* (L. Pfeiffer, 1862), a well-known species that is also associated with the moderate humidity of broad-leaved forests (Figure 12).

#### Hunyadiscus

Taxon classificationAnimaliaPulmonataPlectopylidae

Páll-Gergely gen. n.

http://zoobank.org/AD8518E3-C82B-4E66-A13D-BBB7F90EFC59

##### Type species.

*Hunyadiscus saurini* sp. n.

##### Content.

*andersoni* Blanford, 1869, *saurini* Páll-Gergely, sp. n.

##### Diagnosis.

Shell dextral, body whorl keeled or angulated; protoconch with spiral and radial lines, with the spirals being dominant; palatal plicae slightly sinuate; parietal wall with a single lamella and some additional plicae/denticles anteriorly and/or posteriorly. Internal anatomy unknown. See also Remarks.

##### Differential diagnosis.

*Hunyadiscus* gen. n. differs from all other plectopylid genera by the protoconch sculpture, which is characterized by both spiral and radial lines, the spirals being dominant. Moreover, all species of *Gudeodiscus*, *Halongella* and *Naggsia* gen. n. have a rounded body whorl, which is keeled in *Hunyadiscus* gen. n. The most similar genus to *Hunyadiscus* gen. n. is *Sinicola*, which also possess a keeled (shouldered) body whorl, and usually lacks the apertural fold. See also Table 1.

**Table 1.** Key characters of the shell and genitalia of plectopylid genera possessing ribbed embryonic whorls. One star: see Páll-Gergely et al. 2015b; two stars: see Páll-Gergely et al. 2015a.

Genus	Coiling direction	Apertural fold	Body whorl	Epiphallus	Penial pockets	Protoconch
*Endothyrella*	sinistral or dextral	absent	rounded or keeled	present	whole penial wall	ribbed*
*Sinicola*	dextral	absent (rarely present)	keeled	present	whole penial wall	ribbed
*Gudeodiscus*	dextral	absent or present	rounded	present	apical part	ribbed
*Halongella*	dextral	present	rounded	present	whole penial wall	ribbed**
*Hunyadiscus* gen. n.	dextral	absent	keeled	unknown	unknown	spirally striated and ribbed
*Naggsia* gen. n.	dextral	absent	rounded	absent	absent	ribbed, ribs are wavy, with extremely fine spiral striation
eastern *Sicradiscus*	dextral	absent	keeled	present	whole penial wall	ribbed
western *Sicradiscus*	dextral	present	rounded	present	apical part	ribbed

##### Etymology.

The genus is dedicated to András Hunyadi, Hungarian malacologist and shell collector, who first called the attention of the first author on the necessity of revising the family Plectopylidae. The name *Hunyadiscus* is the combination of the family name Hunyadi and discus (Latin: *disc*), which refers to the shape of the shells.

##### Distribution.

One species (*Hunyadiscus saurini* sp. n.) inhabits southern part of Northern Laos (exact locality unknown), the other species (*Hunyadiscus andersoni*) lives in southern Kachin state (Myanmar), at the bordering Chinese region (Figure 15).

##### Remarks.

Many plectopylid species belonging to the genera *Endoplon*, *Endothyrella*, *Gudeodiscus*, *Halongella*, *Plectopylis*, *Sicradiscus* possess two parietal lamellae (anterior and posterior). Other species, however, possess only a single one. In most cases it is possible to decide that the single lamella is homologous with either the anterior or the posterior lamella, because there are “remains” of the other lamella. For example, in some *Gudeodiscus* species (e.g. *Gudeodiscus multispira* [Möllendorff, 1883]), there are some small denticles in position of the anterior lamella, anterior to the well-developed, curved lamella. This indicates that the curved lamella is homologous with the posterior lamella. In contrast, in the genera *Sicradiscus* and *Endothyrella*, many species have small denticles on the posterior side of the single lamella. This suggests, that the single, well developed lamella is homologous with the anterior lamella (Páll-Gergely and Asami 2016). *Hunyadiscus andersoni* also has two small denticles on the posterior side of the lamella, one above, one below. This suggests that the single lamella of *Hunyadiscus andersoni* is homologous with the anterior lamella. The single lamella of *Hunyadiscus saurini* sp. n. is, on the other hand, probably homologous with the posterior lamella, because it has a strongly curved shape, and has a lower plica positioned anteriorly, which is, when present, situated under the anterior lamella in *Gudeodiscus* species. These hypotheses suggest that the two species of *Hunyadiscus* have remarkably different parietal plication (Fig. 11).

#### Hunyadiscus andersoni

Taxon classificationAnimaliaPulmonataPlectopylidae

(W. Blanford, 1869)

**Figure 3.**
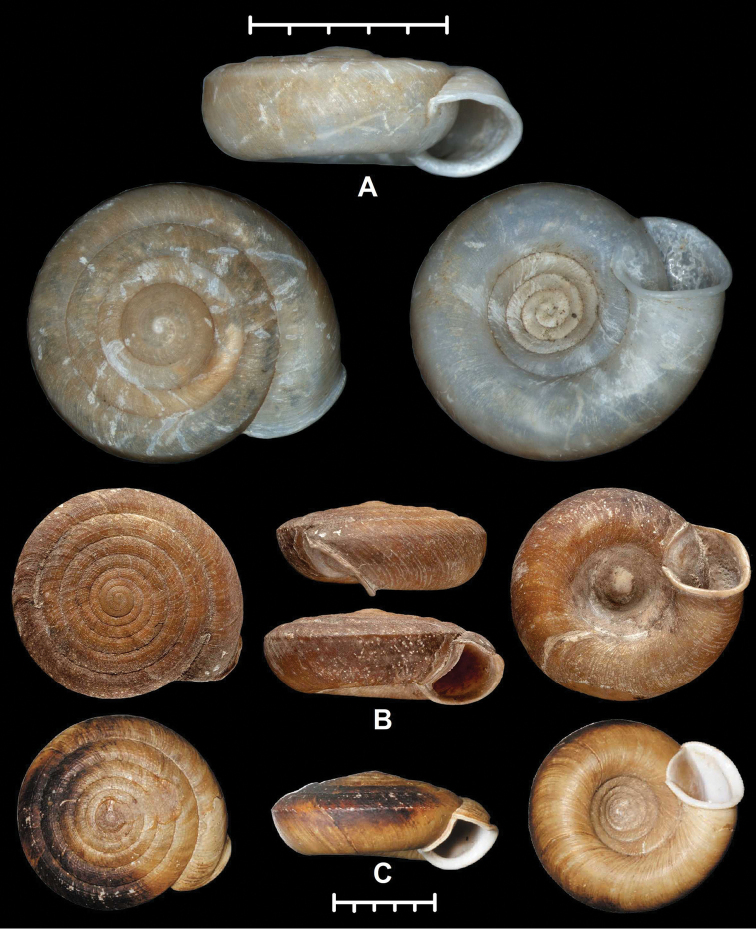
Shells of Plectopylidae species. **A** holotype of *Hunyadiscus saurini* Páll-Gergely sp. n. **B** *Hunyadiscus andersoni* (W. Blanford, 1869) (NHMUK 20130003 **C** Burmah, RBINS 10591. Photos: T. Deli (**C**), J. Harl (**A**) and H. Taylor (**B**). Scales represent 10 mm; upper scale refers to Fig. **A**, lower scale refers to Figs **B** and **C**.

**Figure 4.**
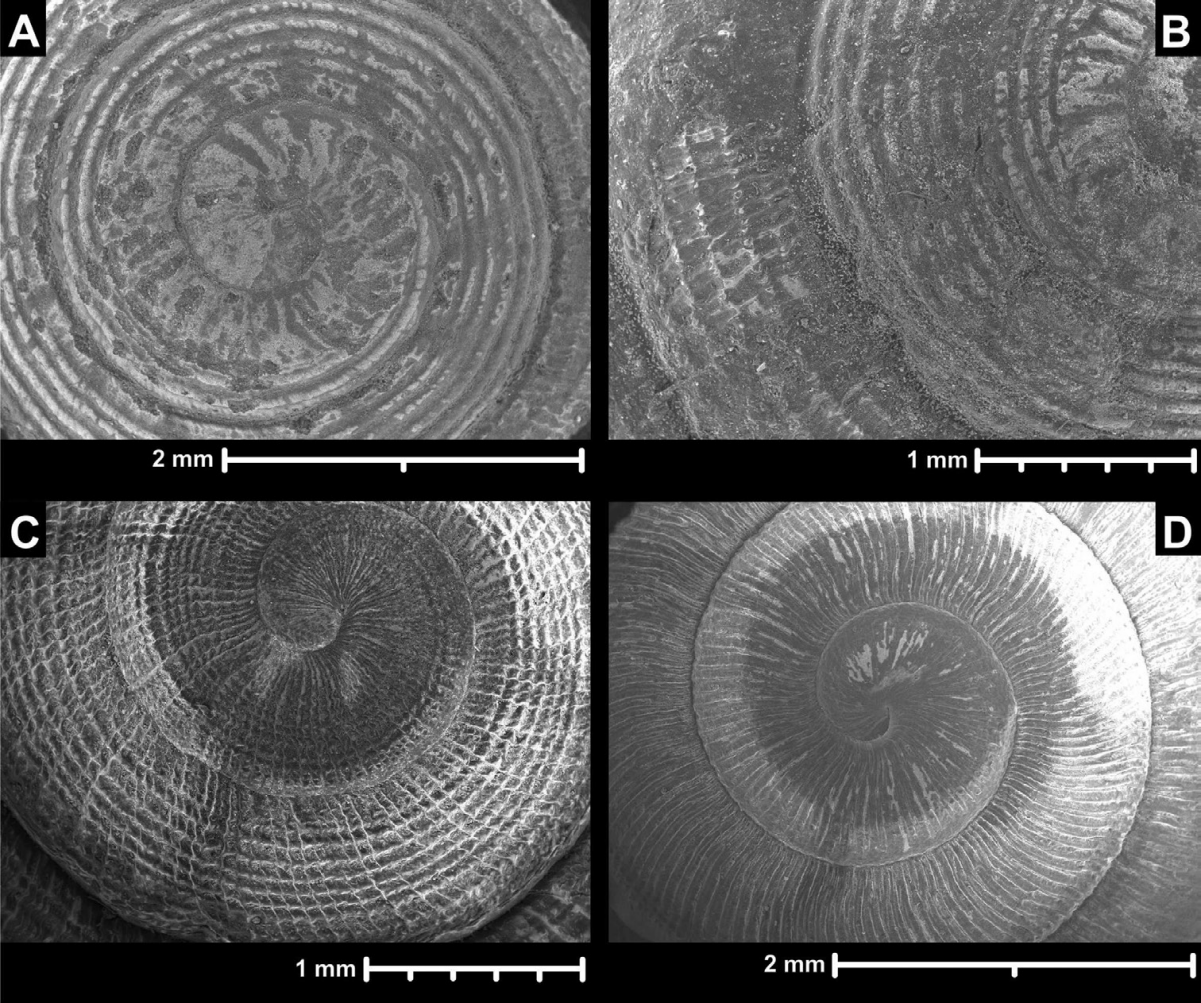
Embryonic whorls of Plectopylidae. **A–B** *Hunyadiscus andersoni* (W. Blanford 1869) (NHMUK 20130003, two different specimens) **C** *Hunyadiscus saurini* Páll-Gergely sp. n. **D** *Naggsia laomontana* (L. Pfeiffer, 1862), same specimen as Fig. 1B. All images by B. Páll-Gergely.

**Figure 5.**
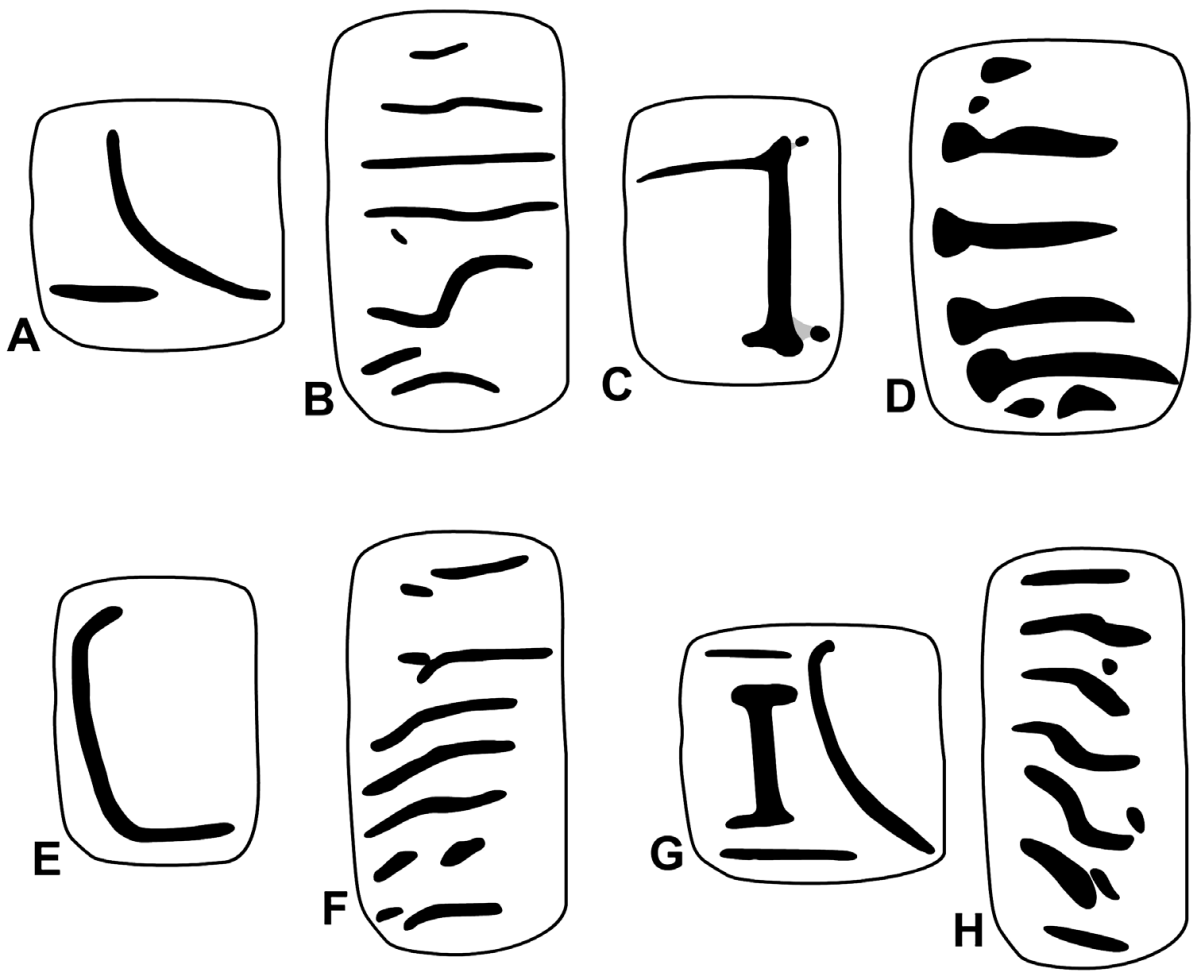
Parietal (**A, C, E, G**) and palatal (**B, D, F, G**) plication of Plectopylidae species. **A–B** *Hunyadiscus saurini* Páll-Gergely sp. n. **C–D** *Hunyadiscus andersoni* (W. Blanford 1869) (**C** SMF 150117 **D** after Gude 1896) **E–F** *Naggsia laomontana* (L. Pfeiffer, 1862), same sample as on Fig. 1B **G–H** Gudeodiscus (Gudeodiscus) messageri raheemi Páll-Gergely & Hunyadi, 2015, same specimen as on Fig. 1C. Figures not to scale. Inner view: **B, F, D**, Outer view: **H**.

Figures 3B–C , 4A–B , 5C–D , 11F

Hunyadiscus andersoni 1869 Helix (Plectopylis) andersoni Blanford, Proceedings of the Zoological Society of London: 448 [Bhamo in regno Avæ et Hoetone in Yunan].
Hunyadiscus andersoni 1874 Helix andersoni, — Hanley and Theobald, Conchologia Indica…: 46, plate 112, figs 8–9 [Bhamo, and Hoetone in Yunan] (1870–1876).
Hunyadiscus andersoni 1875 Helix (Plectopylis) andersoni, — Godwin-Austen, Proceedings of the Zoological Society of London: 612, Plate 74, fig. 9.
Hunyadiscus andersoni 1885 Plectopylis Andersoni, — Möllendorff, Jahrbücher der Deutschen Malakozoologischen Gesellschaft, 12: 389 [“bei Bhamo in Oberbirma”, “»Hoitone« in der chinesischen Provinz Yünnan”].
Hunyadiscus andersoni 1886 Plectopylis Andersoni, — Möllendorff, Jahrbücher der Deutschen Malakozoologischen Gesellschaft, 13: 188.
Hunyadiscus andersoni 1887 Helix andersoni, — Tryon, Manual of Conchology. 2 (3): 161, Plate 34, fig. 71; Plate 35, figs 74–75 [Bhamo, in Ava; Hoetone, In Yunan].
Hunyadiscus andersoni 1889 Helix (Plectopylis) Andersoni, — Tapparone Canefri, Annali del Museo Civico di Storia Naturale di Genova, 2a (7): 47 (=323). [“Catcin di Pun-Can, Cimfó, Monti Est di Bhamó”, “Bhamó, Hoetone”]
Hunyadiscus andersoni 1896 Plectopylis andersoni, — Gude, Science Gossip, 3: 154, figs 17a–c [Near Bhamo and Ava, in Upper Burma and on the Yunnan-frontier].
Hunyadiscus andersoni 1899a Plectopylis (Chersaecia) andersoni, — Gude, Science Gossip, 6: 148.
Hunyadiscus andersoni 1899b Plectopylis (Chersaecia) andersoni, — Gude, Science Gossip, 6: 175.
Hunyadiscus andersoni 1914 Plectopylis (Chersaecia) andersoni, — Gude, The Fauna of British India including Ceylon and Burma. Mollusca II. (Trochomorphidae – Janellidae), 2: 73, 114, figs 55a–c.
Hunyadiscus andersoni 2013 Chersaecia andersoni, — Páll-Gergely and Hunyadi, Archiv für Molluskenkunde 142 (1): 7, figs 14–15.

##### Types.

According to Dance (1986) the collection of Blanford is deposited in the British Museum (now: Natural History Museum, London). In the type collection of the NHM we did not find syntypes, but there is a sample (NHMUK 1906.02.02.364) which is labelled as being collected from Bhamo, one of the type localities. This sample may represent the type lot.

##### Museum material examined.

Yünan, Slg. Bosch, ex H. Rolle (1 juv.), SMF 172066; alte Schau-Slg/2, SMF 150117; no locality information (alte Schau-Slg.), SMF 150117/2; Upper Indwadi, NHMUK 1888.12.04.1561/2; Burma, coll. A. S. Kennard, ex Gude, NHMUK/2; Bhamo, Upper Burma, NHMUK 1906.02.02.364/5; Khakhyan Hills, Burmah, coll. Godwin-Austen, NHMUK/3; Bhamo, NHMUK/1.

##### Diagnosis.

A very large, discoid, angulated species with elevated, sharp callus and spirally striated protoconch.

##### Description.

Shell flat, angulated, light brown or corneous; ventral side of the body whorl keeled around the moderately wide, very deep umbilicus; protoconch spirally striated, radial ribs are mostly visible on its first whorl only; teleoconch equally ornamented with fine ribbing and spiral striae, resulting in rough, irregular reticulated surface on the dorsal side; ventral side also reticulated, but much weaker than the dorsal surface; 7.5–8.5 slowly increasing whorls separated by shallow suture; near the sutures the riblets sometimes supported with fine folds of the periostracum; aperture rounded, with white, slightly expanded and thickened apertural rim; callus slightly elevated, sharp, slightly S-shaped and forms two canals upon junction with the lip.

The parietal side was examined in one specimen (SMF 150117), whereas the palatal plicae is examined in specimens of the SMF and the NHM. Parietal wall with one curved horizontal lamella with occasionally an elongated upper plica, which is in contact with the lamella; there are two small denticles on the posterior side of the lamella, these are in weak contact with the lamella; palatal side with eight horizontal plicae, first near the suture is small, the second is even smaller; the last also short and close to the lower suture and the penultimate resembles the second denticle; remaining four plicae between the first and last two are long and slim.

##### Measurements

(in mm). D = 24.6–27.2, H = 10.9–11.4 (n = 2, RBINS I. G. 10591, Burmah).

##### Differential diagnosis.

This species differs from large Chinese *Gudeodiscus* species (and *Naggsia laomontana*) by the keeled margin of the shells. It is much larger than all *Sinicola* species. The largest *Sinicola* species, *Sinicola fimbriosa* (Martens, 1875) does not have a callus and has a stronger apertural margin.

##### Distribution.

The species is known from Northern Burma and Western Yunnan. Hoetone (Hutung Village) and Bhamo are located in Kachin Provinces, whereas Ava is in Mandalay Province (all in Burma/Myanmar). The Kakhyen Hills are situated on the Chinese (Yunnan) and Burmese (Kachin) border.

#### Hunyadiscus saurini

Taxon classificationAnimaliaPulmonataPlectopylidae

Páll-Gergely sp. n.

http://zoobank.org/EF0B1D36-1FBB-49A5-972C-89E6BCA3FBCB

Figures 3A , 4C , 5A–B , 11A

Hunyadiscus saurini 1953 Plectopylis laomontana, Saurin, *Journal de Conchyliologie*, 93 (4), 113.

##### Type material.

Laos, Pa Hia (Ancienne province Tran Ninh), Coll. Saurin, MNHN 24947 (holotype), MNHN 24948/7 paratypes + 5 juvenile shells (also paratypes), HNHM 97470/2 paratypes; Laos, Pah Xieng Tong, Pa Hia, Prov. Tran Ninh, Coll. Saurin, MNHN 249479/1 paratype + 1 juvenile shell (also paratype, protoconch figured: Fig. 4C); Laos, Pa Ka Tai, Prov. Tran Ninh, Coll. Saurin, MNHN 24950/5 paratypes, HNHM 97471/1 paratype; Laos, Pa Xieng Tong, Pa Hia, Prov. Tran Ninh, Coll. Saurin MNHN 24951/3 paratypes; Laos, Pa Hia (Tran Ninh), Coll. Saurin, MNHN 24952/1 juvenile shell (paratype).

**Figure 6.**
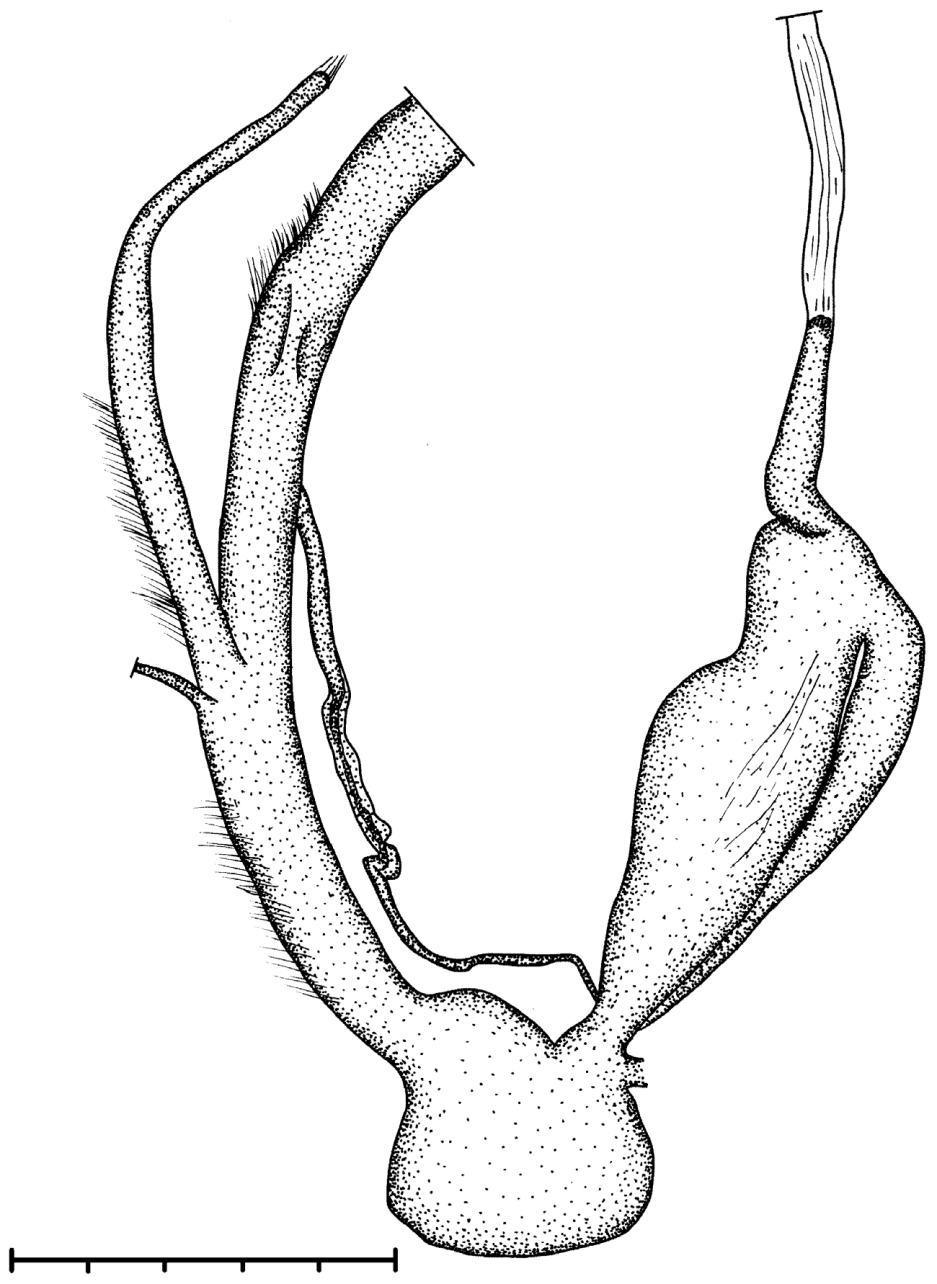
Reproductive anatomy of Gudeodiscus (Gudeodiscus) messageri raheemi Páll-Gergely & Hunyadi, 2015, same specimen as on Fig. 1C and Fig. 2B. Scale represents 5 mm.

##### Diagnosis.

A dextral, medium-sized or large species with a relatively sharp upper keel and a blunt lower keel on the body whorl. On the parietal wall there is a single oblique lamella with a horizontal plica below it.

##### Description.

The shell is yellowish or corneous (the type material consists mainly of weathered shells). The protoconch is very large, with regular riblets and spiral lines; the radial and spiral lines are approximately of the same strength. The 5.25–6 whorls are separated by a shallow suture. The umbilicus is wide but moderately deep. The body whorl has a prominent upper keel and a less conspicuous lower keel. The apertural margin is slightly thickened. The parietal callus is blunt, not well developed and is only clearly apparent in older specimens.

Three shells were opened. On the parietal wall there is a single curved lamella that is oblique, its upper end situated much more anteriorly than the lower end. A short, but thick vertical plica is situated below and anteriorly of the lamella. On the palatal wall there are six more-or-less parallel plicae, with some additional short plicae. The most prominent additional plica is situated above the posterior end of the last plica. The fifth plica is usually S-shaped.

##### Measurements

(in mm). D= 16.3–21.3, H= 6.7–8.6. (n=4, shells from different samples).

##### Differential diagnosis.

*Hunyadiscus saurini* sp. n. is smaller than *Hunyadiscus andersoni*, its keel is situated higher (this results a more angular body whorl), has weaker parietal callus, a lower horizontal plica on the parietal wall which is absent in *Hunyadiscus andersoni*. *Naggsia laomontana* has a rounded body whorl and weaker spiral striation on its protoconch.

##### Etymology.

The species is named in honour of the French geologist and malacologist Edmond Saurin (1904–1977) who collected it.

##### Type locality.

Laos, Pa Hia (Ancienne province Tran Ninh).

##### Distribution.

This species is known only from Southern Laos.

##### Remarks.

This new species shows considerable diversity in terms of shell size. However, the other shell characters are stable within and between samples.

The village “Pa Hia” or “Pah Hia” is located 100 km south from Xieng-Khouang, the capital of Tran Ninh Province (see Saurin 1953: 113). However, the exact locality could not be determined (Nordsieck 2002, Páll-Gergely 2014, Páll-Gergely 2015c). A geological report (Marutani 2006), mentioned the name “Ban Namthong” in brackets after “Pa Hia”. The two names are probably identical, but the origin of this information could not be traced. Marutani (2006) gave the following GPS coordinates for Ban Namthong: 19.05000°N, 103.28330°E. This location is situated approximately 75 km southwest from Xiangkhoang city. Google Earth placed “Ban Namthong” 7.6 km in southwest direction (18° 59'N, 103° 16'E), which agrees with the 1:50.000 map printed by the National Geographic Directorate, Vietnam, in 1965. We provisionally identify the village Pah Hia with Ban Namthong, because we could not locate the name Pah Hia on the maps available to us.

#### Naggsia

Taxon classificationAnimaliaPulmonataPlectopylidae

Páll-Gergely & Muratov gen. n.

http://zoobank.org/D8CDB123-6382-462E-A66E-ABFD7C838CA3

##### Type species.

*Helix laomontana* L. Pfeiffer, 1862.

##### Content.

*Naggsia laomontana* (L. Pfeiffer, 1862).

##### Diagnosis.

Shell flat, widely umbilicated, body whorl rounded; protoconch with dense, regular, slightly waved ribs with extremely fine spiral striation. Epiphallus absent, diverticulum and gametolytic sac are both very short, but diverticulum is still shorter than the gametolytic sac. Cusp of central tooth missing, only basal plate of central tooth present. Marginals bicuspid.

##### Differential diagnosis.

*Naggsia* gen. n. differs from the genera having ribbed embryonic whorls (*Endothyrella*, *Gudeodiscus*, *Halongella*, *Sicradiscus*, *Sinicola*) by the absence of an epiphallus and the presence of a very short diverticulum. Moreover, although the protoconch of *Naggsia* gen. n. is ribbed, the ribs (radial lines) are not straight, as in the other genera, but are somewhat wavy. *Naggsia* gen. n. differs from the genera without a ribbed protoconch (*Chersaecia*, *Endoplon*, *Plectopylis*) by the presence of regular, slightly waved ribs on the embryonic whorls. The latter genera are insufficiently known anatomically. *Plectopylis bensoni* Gude, 1914 (mentioned as *Plectopylis achatina* Pfeiffer, 1845) and *Plectopylis cyclaspis* Benson, 1859 have a well-developed epiphallus (Stoliczka 1871). Interestingly, these *Plectopylis* species have a relatively short and thickened diverticulum, which is somewhat similar to that of *Naggsia laomontana*. Similarly to *Naggsia laomontana*, *Chersaecia simplex* Solem, 1966 lacks the epiphallus (see Solem 1966), but it at has no diverticulum, which is well developed in *Naggsia*. See differential diagnosis under *Hunyadiscus* and Table 1.

##### Etymology.

The new genus is dedicated to Fred Naggs (NHM) in acknowledgement of his help with our studies on the Plectopylidae.

##### Distribution.

Northern Laos (Figure 15).

##### Remarks.

The anatomy of *Naggsia laomontana* is rather similar to that of *Plectopylis* in the short diverticulum and to *Chersaecia* in the absence of epiphallus. The shell characters, namely the ribbed protoconch and the reduced parietal plication is similar to the genera *Endothyrella*, *Gudeodiscus*, *Halongella*, *Sicradiscus* and *Sinicola*. The radula of *Naggsia laomontana* shows similarities with those of Gudeodiscus (Veludiscus), *Halongella* and *Plectopylis* (see Stoliczka 1871, Páll-Gergely et al. 2015a) in terms of the small central tooth and the simple marginals. In contrast, the central tooth of *Endothyrella*, Gudeodiscus (Gudeodiscus), *Sicradiscus* and *Sinicola* species is large (as large as or larger than the ectocones of the first laterals), (Páll-Gergely et al. 2015a, 2015b), and the marginals are tricuspid or even quadricuspid. Before examining ethanol-preserved *Naggsia laomontana* specimens, we considered grouping the plectopylid genera into two tribes, namely one with a ribbed, and another with a smooth or granulated protoconch. The examination of *Naggsia laomontana* revealed that the character states considered to be primarily important do not allow the placement of *Naggsia* gen. n. in any of the two groups, and that the character states rather show a mosaic structure across genera.

#### Naggsia laomontana

Taxon classificationAnimaliaPulmonataPlectopylidae

(L. Pfeiffer, 1862)

Figures 1A–B , 2A , 4D , 5E–F , 7 , 8 , 9A–C , 10

**Figure 7.**
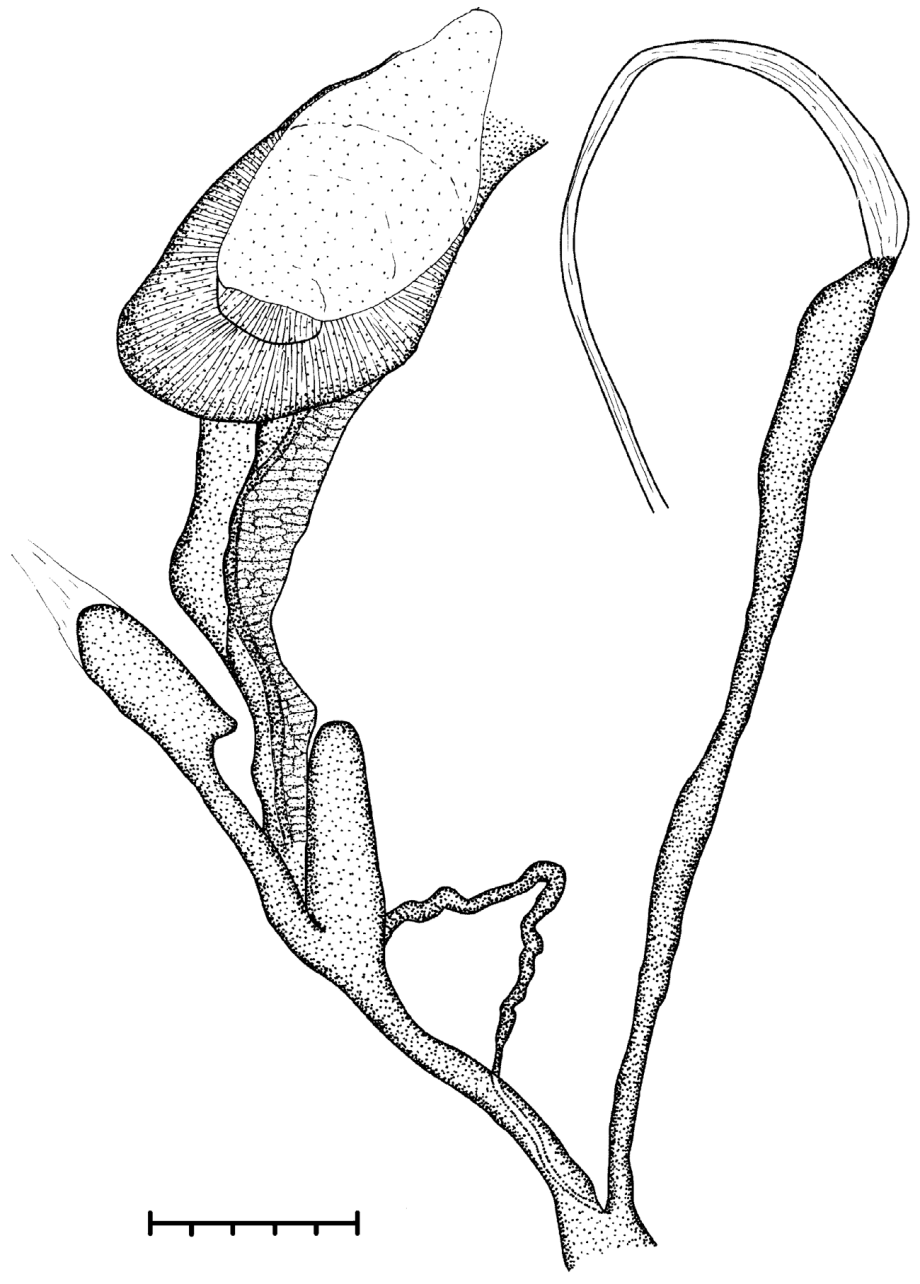
Reproductive anatomy of *Naggsia laomontana* (L. Pfeiffer, 1862), 16L06, spec.1. Scale represents 5 mm.

**Figure 8.**
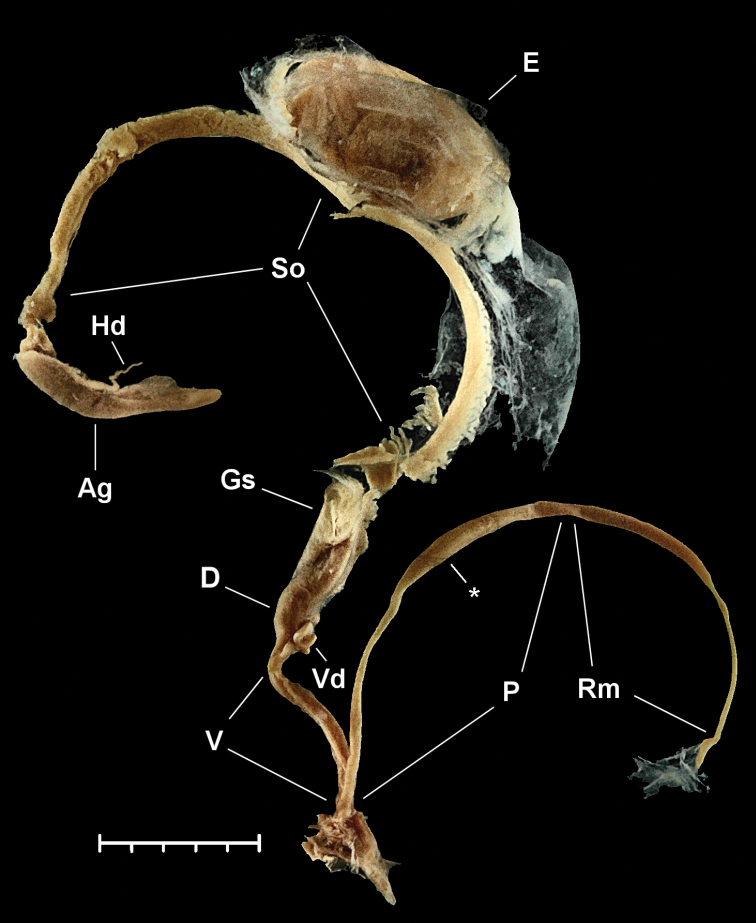
Photo of the reproductive anatomy of *Naggsia laomontana* (L. Pfeiffer, 1862), 16L06, spec.2. Abbreviations: Ag: albumin gland; D: diverticulum; E: embryo in the uterus (one out of five); Gs: gametolytic sac; Hd: hermaphroditic duct; P: penis; Rm: retractor muscle; So: spermoviduct; V: vagina with vas deferens alongside; Vd: coiled portion of vas deferens. Asterix indicates the place until where vas deferens could be traced back. Scale represents 5 mm.

**Figure 9.**
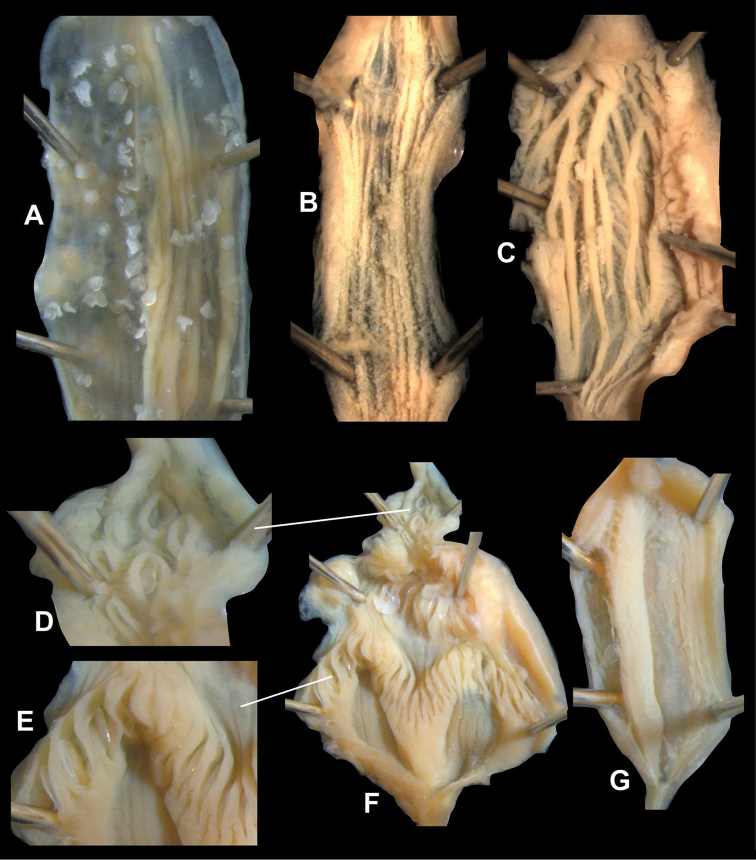
Inner walls of reproductive organs of Plectopylidae. **A–C** *Naggsia laomontana* (L. Pfeiffer, 1862) (same specimen as on Fig. 7); Gudeodiscus (Gudeodiscus) messageri raheemi Páll-Gergely & Hunyadi, 2015 (same specimen as on Fig. 1C). **A, E** penis **B, G** epiphallus **C** diverticulum **D** penial caecum **F** penis and penial caecum. Figures not to scale. All images: B. Páll-Gergely.

**Figure 10.**
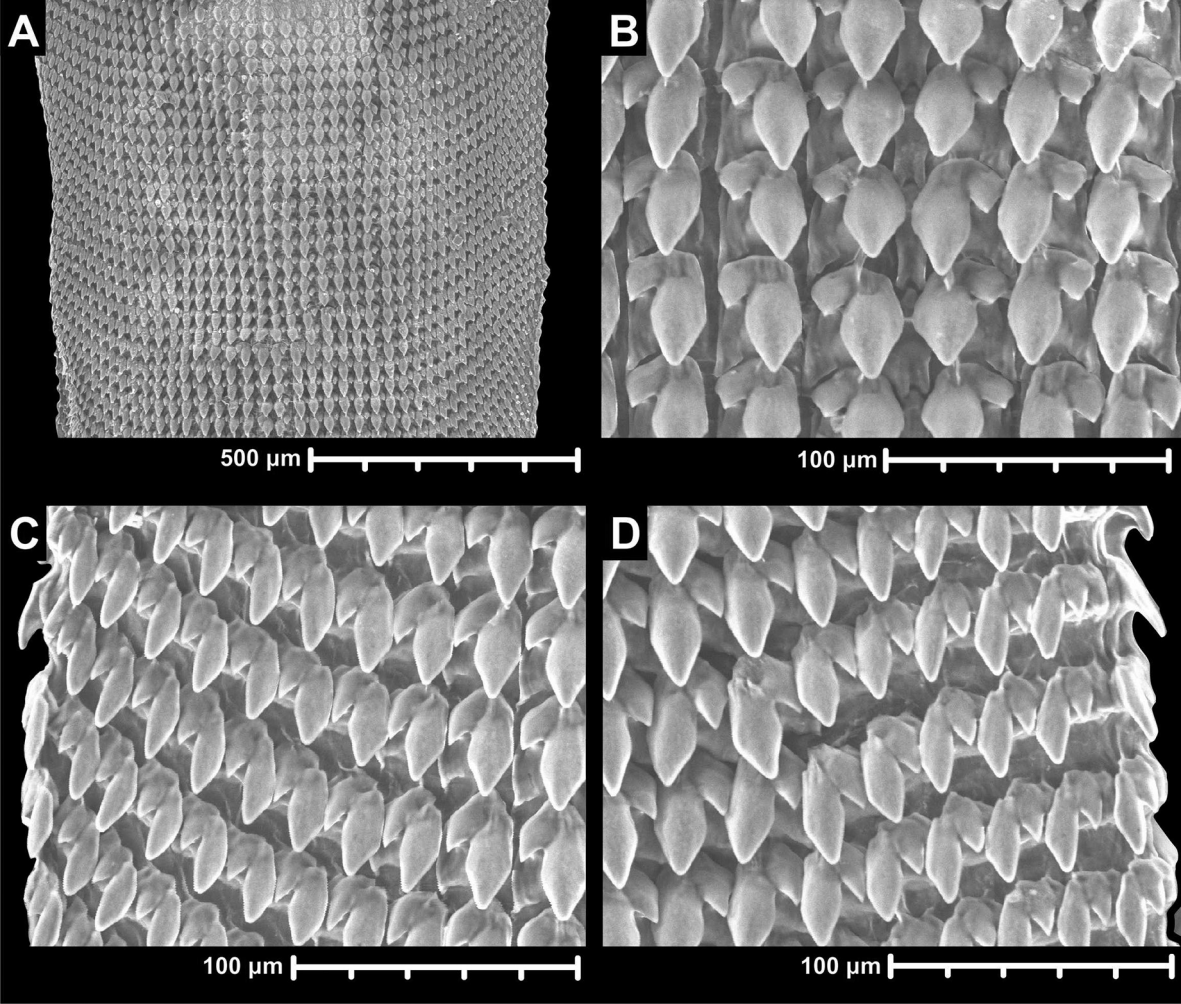
Radula of *Naggsia laomontana* (L. Pfeiffer, 1862). **A** anterior-posterior middle section of the Radula **B** central and first two lateral teeth **C–D** marginals. Sample: 16L06.

Naggsia laomontana 1862 Helix laomontana L. Pfeiffer, Proceedings of the Zoological Society of London, 272, Plate 36, figs 9–10 [Lao Mountains, Camboja].
Naggsia laomontana 1863 Helix laomontana, — L. Pfeiffer, Novitates Conchologicae 2: 216, Plate 57, figs 7–9.
Naggsia laomontana 1868 Helix laomontana, — L. Pfeiffer, Monographia Heliceorum Viventium...: 394.
Naggsia laomontana 1875b Helix (Plectopylis) laomontana, — Godwin-Austen, Proceedings of the Zoological Society of London: 612.
Naggsia laomontana 1887 Helix laomontana, — Tryon, Manual of Conchology, 2 (3): 160, plate 34, figs 60–62.
Naggsia laomontana 1897a Plectopylis laomontana, — Gude, Science Gossip, 3: 245, figs 36a–c.
Naggsia laomontana 1899a Plectopylis (Chersaecia) laomontana, — Gude, Science Gossip, 6: 148.
Naggsia laomontana 1899b Plectopylis (Chersaecia) laomontana, — Gude, Science Gossip, 6: 175.
Naggsia laomontana 1914 Plectopylis (Chersaecia) laomontana, — Gude, The Fauna of British India including Ceylon and Burma. Mollusca.−II. (Trochomorphidae-Janellidae): 73.
Naggsia laomontana 1920 Plectopylis laomontana, — Gude, Proceedings of the Malacological Society of London, 14: 62, fig. 1.
Naggsia laomontana 2013 Chersaecia laomontana, — Páll-Gergely and Hunyadi, Archiv für Molluskenkunde 142 (1): 7–8.

##### Types examined.

Cambodia, NHMUK 20130004 (3 syntypes).

##### Museum material examined.

Laos, Luang Prabang (alte Schau-slg.), SMF 150121/2; Laos (Siam), Luang Prabang, ex Möllendorff, SMF 294866/3; Laos, Luang Prabang slg. Dosch ex H. Rolle, SMF 172067/1; Laos, Luang Prabang, SMF 150122/4; Cambodia, slg. Dosch ex H. Rolle ex Sowerby ex Fulton, SMF 172068/3; Laos, Luang Prabang, Französ. Hinterindien, C. Boettger 1904/43, SMF 102819/3; Cambodia, NHMW 342232/2; Laos, Lao Mountains, Altonaer Museum, coll. Semper, O. ex Cuming, ZMH 45901/2; Cambodge, coll. Achat Lallé, 1870, MNHN 2012-2506/1; Louang Prabang, MNHN 2012-2507/1; Cambodge, coll. Deshayes in coll. Crosse, MNHN 2012-2508/1; Laos, coll. Denis, MNHN 2012-2509/1; Louang Prabang, coll. Morgan, MNHN 2012-2510/2; Mts. Lao, Cambodja, MNHN 2012-2511/1; Louang Prabang, coll. Letellier 1949, MNHN 2012-2512/1; Louang Prabang (Laos), coll. Morlet-Fischer, MNHN 2012-2513/4; Louang Prabang (Laos), coll. Staadt 1969, MNHN 2012-2514/2; Louang Prabang (Laos), coll. Morlet-Fischer, MNHN 2012-2515/2; China, coll. Salisbury ex Beddome (also Canon Hoisley coll., 1918), NHMUK 20110363; Cambojia, Mr. Mouhot, Lao Mountains, NHMUK/3; Siam, Lao Mountains, coll. Godwin-Austen, NHMUK/2; India, NHMUK/2; Camboja, NHMUK/1; Laos, Luang Prabang, coll. Möllendorff, NHMW 7285/2; Tonkin, Prabang, coll. Gerstenbrandt, NHMW 8467/2; Laos. Luang Prabang, coll. Möllendorff, NHMW 40181/6; Cambodia, NHMW 34232/2;

##### New material examined.

Laos, Luang Prabang Province, Ban Pak Ou, Nam Wu (opposite side of Ban Pak Ou), 364 m 20°03.48276'N, 102°12.79912'E, leg. Ohara, K. 13.10.2006., PGB/5; Laos, Tad Kuangsi Waterfall, about 20 km SW of Luang Prabang, 19°43'02.97”N 101°59'38.68”E., leg. Reischütz, A., February 2010., RE/3; Laos, Tad Kuangsi Waterfall, about 20 km SW of Luang Prabang, 19°43'02.97”N 101°59'38.68”E, leg. Theisl, T. April 2009., RE/3+2 juv.; Laos, Luang Prabang Province, Tad Kuangsi Xi (Waterfall), 466 m, 19°44.96071'N, 101°59.49286'E, leg. Ohara, K. 14.10.2006., PGB/1; **16L06** Laos, Luang Prabang Province, ca. 7 km S of Luang Prabang, Near Tad Thong waterfall, 431 m a.s.l., 19°50.064'N, 102°07.755'E, leg. A. Abdou, I.V. Muratov, 3.11.2006., MNHN 2012-27057/45 shells + anatomically examined specimens (Figs 2A, 7–8, 9A–C, 10); **39L06** Laos, Luang Prabang Province, ca. 5 km SE of Luang Prabang, ca. 1.5 km NE of Ban Lak Sip, Phou Xuang mountain, 640 m a.s.l., 19°51.605'N, 101°11.081'E, leg. A. Abdou, I.V. Muratov, 24.11.2006., MNHN 2012-27057/20 shells (some of them broken/juvenile); **42L06** Laos, Luang Prabang Province, ca. 22 km SW of Luang Prabang, Kuang Si waterfall, 482 m a.s.l., 19°44.966'N, 101°59.496'E, leg. A. Abdou, I.V. Muratov, 28.11.2006., MNHN 2012-27057/14 (some of them broken/juvenile).

##### Diagnosis.

A dextral, medium-sized or large species with a rounded body whorl, and no apertural fold. On the parietal wall there is a single curved lamella.

##### Description.

The yellowish, sometimes pink or light brown shell is dextral, almost flat with the apex slightly elevated. The 5.5–6 whorls are separated by a moderately deep suture. The protoconch is very densely, regularly ribbed, with extremely fine spiral lines across the ribs. The teleoconch is irregularly ribbed; the space between the ribs is greater than on the protoconch. The lip is only slightly thickened and reflexed. There is an elevated, but blunt parietal callus, which has two shallow channels at the meeting point with the parietal part of the lip.

Four specimens were opened. On the parietal wall there is a single curved lamella without additional plicae. On the palatal wall there are seven horizontal plicae. The first, (situated near the suture) is short, undivided, not inclined, sometimes having a short denticle slightly lower than its posterior end. The second plica is slightly indented in place opposing the parietal curved lamella just before it becomes dichotomously bifurcated posteriorly, with its lower posterior arm slightly inclined away from the suture. The third, fourth and fifth exhibit an increasing tendency to be divided opposing the parietal lamella and inclined posteriorly away from the suture. The sixth is strongly, equally divided, having both parts equally inclined posteriorly away from the suture. The last one, unequally divided, consists of a long, not inclined anterior part and short, inclined posterior part.

##### Differential diagnosis.

*Naggsia laomontana* resembles *Gudeodiscus* species in having the single parietal lamella, rounded body whorl and densely ribbed protoconch. The protoconch of *Naggsia laomontana* however reveals a unique surface structure, the riblets are comprised of slight waves that do not stand as regularly as those of *Gudeodiscus*. *Gudeodiscus* species that usually have a somewhat elevated spire, more whorls, two horizontal plicae in front of the parietal lamella, and simple (undivided) palatal plicae.

##### Measurements

(in mm). D= 28.3–32, H= 8.8–9.1 (n=3, syntypes); D= 18.6–21, H= 6.4–7.5 (n=5, specimens from Laos).

##### Characters of the genital structure

(Figs 7–8, 9A–C). Two specimens were anatomically examined (sample 16L06). The right retractor muscle crosses between the penis and vagina.

Penis long, its distal part is more slender than the proximal part, internally with 5–6 longitudinal folds aligned next to each other; only one of the folds reach the proximal end of the penis, the others are shorter; the penial wall (outside of the folded area) is wrinkled; the wrinkles are stronger near the distal end of the penis; many small, flat, lenticular calcareous granules were found in the penis lumen; epiphallic differentiation was not detected; retractor muscle slightly thinner than penis, shorter than it and connected to the apical end of penis; vagina shorter than half of penis; vas deferens has thick coiled portion just after coming out of spermoviduct, connects to vaginal wall and forms part of penial wall, reaching the middle of the proximal part of penis; diverticulum short, oval, gametolytic sac with relatively thick, cylindrical stalk and thickened, rather quadrangular sac; there were five, well-developed embryos in the uterus.

##### Radula

(Figure 10). Radula elongated, but not very slender; the basal plates of the centrals are present, but their cusps are absent; the teeth are arranged in rows; each row contains 18–19 teeth, the first nine are laterals, the remaining are marginals, but it is rather difficult to decide which teeth are the last laterals and the first marginals; lateral teeth stand in straight rows which are perpendicular to the central column; marginals stand in anteriorly pointed, slightly oblique rows; endocones of laterals are rhomboid, rather blunt; ectocones are small, pointed, triangular; endocones of marginals are slender ovoid, blunt; the ectocones are small, pointed, triangular.

##### Distribution.

The species was described from “Lao Mountains, Camboja”. We have seen material with more detailed geographical data only from the central part of Northern Laos (around Luang Prabang). In the collection of the Natural History Museum London, a single shell of *Chersaecia laomontana* is present with the locality “China” (NHMUK 20110363, Salisbury Collection Ex Beddome Ex Canon Hoisley coll. 1918; see Páll-Gergely and Hunyadi 2013). Its occurrence in China is not verified and the locality is possibly wrong.

##### Ecology and behaviour.

This species can be found in primary or old secondary broad-leaved forests, but it inhabits some peculiar habitats as well. It can survive droughts as well as periodical floods and can be found in large numbers near waterfalls (Kuang Si waterfall, for example, is one very popular collecting spot) (Figure 13). Unlike most terrestrial snails that start to crawl when placed in water, snails of this species, when under water, retract deep into the shell, which is probably an adaptation that helps to survive frequent floods.

**Figure 11.**
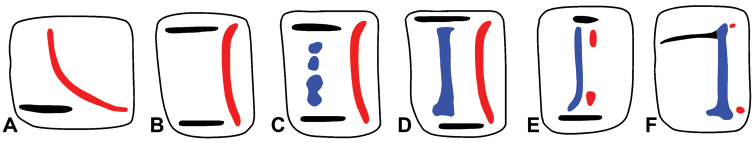
Parietal plication of Plectopylidae species (diagrammatic figures). **A** *Hunyadiscus saurini* sp. n. **B–E** main character states of *Endothyrella*, *Gudeodiscus*, *Halongella*, *Sicradiscus* and *Sinicola* (mainly after Páll-Gergely and Hunyadi 2013 and Páll-Gergely et al. 2015b and Páll-Gergely and Asami 2016) **F** *Hunyadiscus andersoni* (W. Blanford 1869). Blue colour indicates the anterior lamella and its homologous structures; red colour indicates the posterior lamella and its homologous structures.

**Figure 12.**
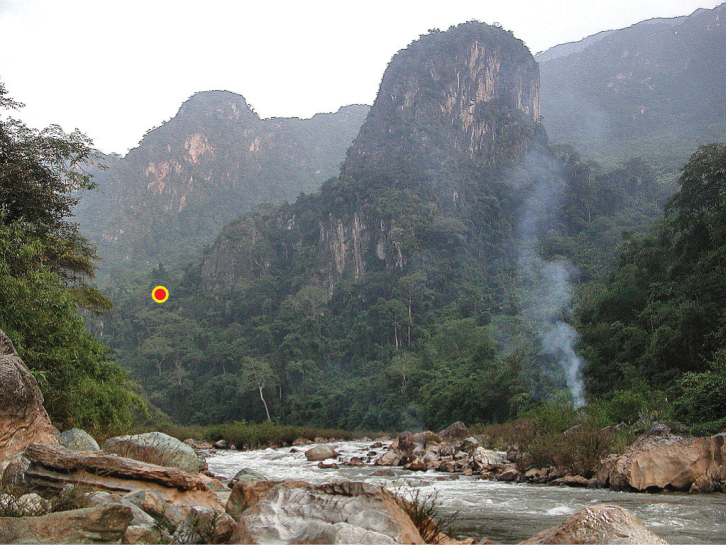
Habitat of Gudeodiscus (Gudeodiscus) messageri raheemi Páll-Gergely & Hunyadi, 2015. The left side of Nam Khan ca. 18 km SE of Muang Xiang Ngeun. The red dot indicates the approximate place where the specimens were collected. Photo: Igor Muratov.

**Figure 13.**
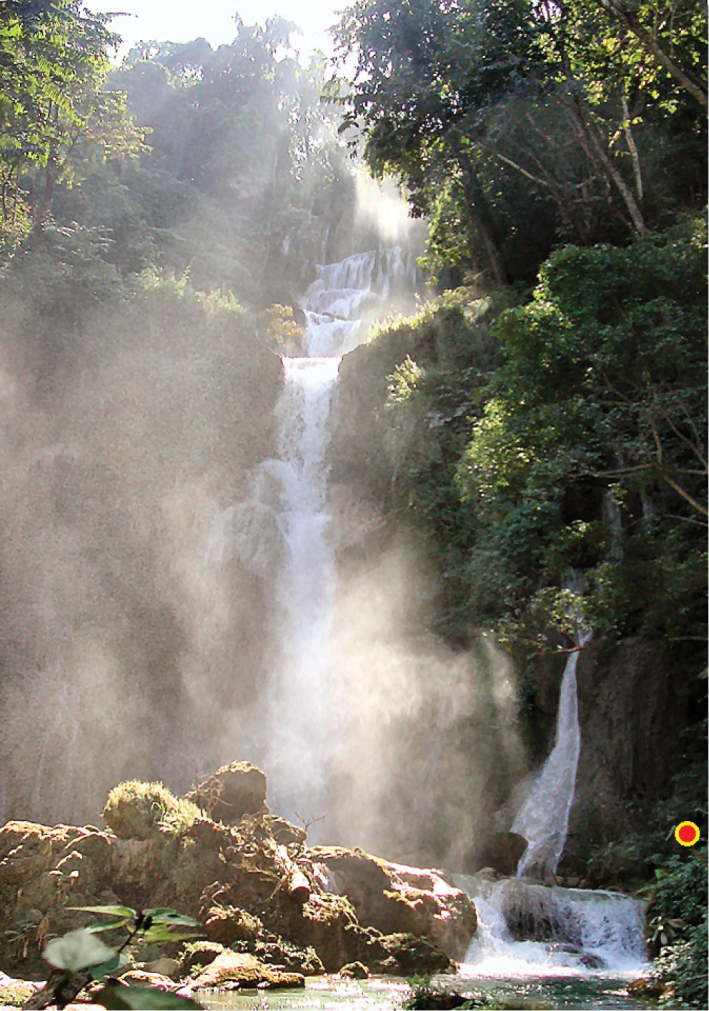
Habitat of *Naggsia laomontana* (L. Pfeiffer, 1862). Kuang Si waterfall. The red dot indicates the exact place where the specimens were collected. Photo: Igor Muratov.

**Figure 14.**
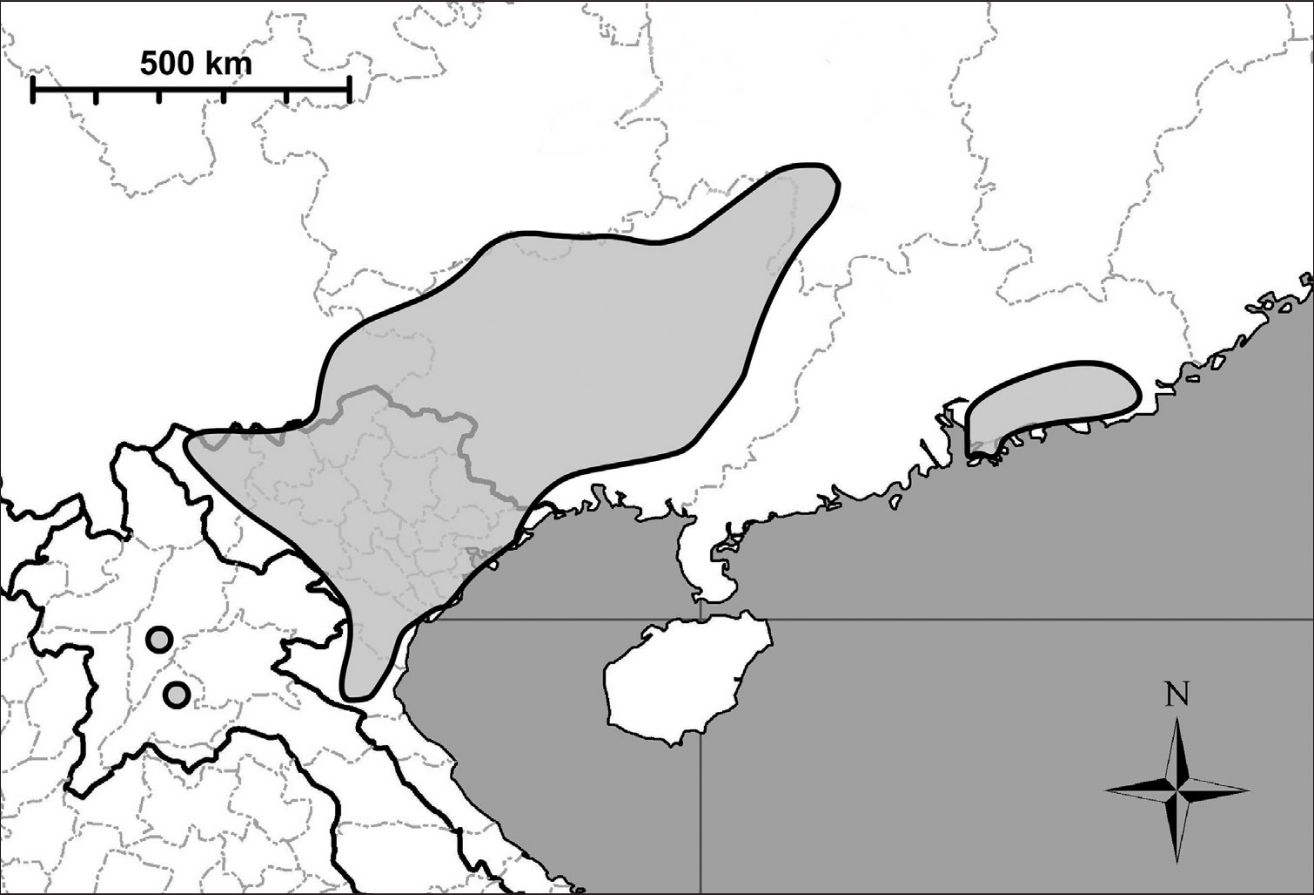
Map showing the distribution of *Gudeodiscus* Páll-Gergely, 2013. The small circles in Laos indicates the new localities of Gudeodiscus (Gudeodiscus) messageri raheemi Páll-Gergely & Hunyadi, 2015. After Páll-Gergely and Hunyadi (2013) and Páll-Gergely et al. 2015a.

**Figure 15.**
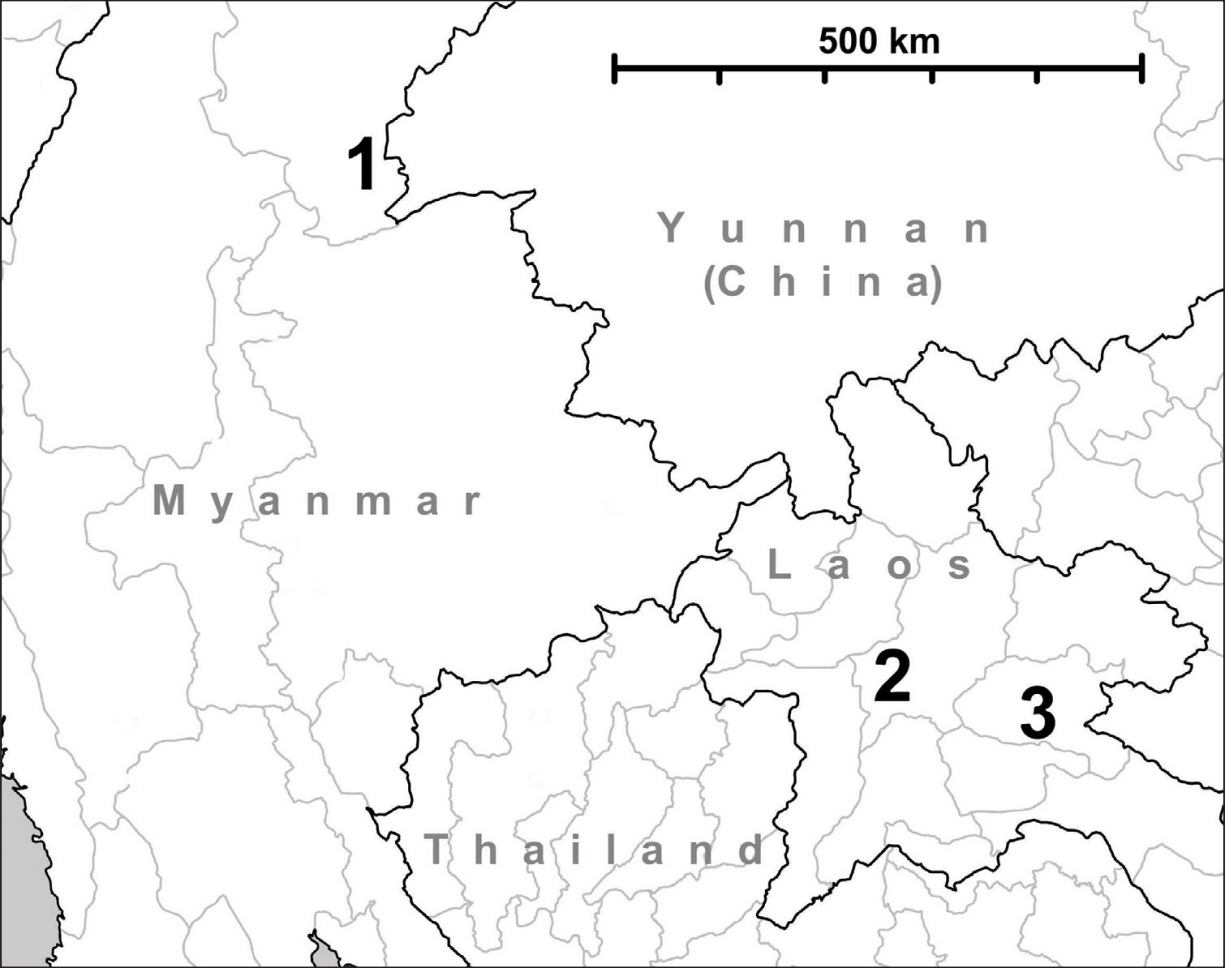
Map showing the distribution of *Hunyadiscus andersoni* (W. Blanford, 1869) (**1**), *Naggsia laomontana* (L. Pfeiffer, 1862) (**2**) and *Hunyadiscus saurini* Páll-Gergely, sp. n. (**3**). For the accuracy of location no. 3. see under *Hunyadiscus saurini*.

## Supplementary Material

XML Treatment for Gudeodiscus

XML Treatment for Gudeodiscus

XML Treatment for Gudeodiscus (Gudeodiscus) messageri raheemi

XML Treatment for Hunyadiscus

XML Treatment for Hunyadiscus andersoni

XML Treatment for Hunyadiscus saurini

XML Treatment for Naggsia

XML Treatment for Naggsia laomontana
